# Physical Therapy Rehabilitation for a Chronic Alcoholic Patient With Loculated Pleural Effusion

**DOI:** 10.7759/cureus.30368

**Published:** 2022-10-16

**Authors:** Nidhi Tiwari, Ruhi Kumbhare, Rashmi R Walke

**Affiliations:** 1 Physical Therapy, Ravi Nair Physiotherapy College, Datta Meghe Institute of Medical Sciences, Wardha, IND; 2 Cardiorespiratory Physiotherapy, Ravi Nair Physiotherapy College, Datta Meghe Institute of Medical Sciences, Wardha, IND; 3 Physiotherapy, Ravi Nair Physiotherapy College, Datta Meghe Institute of Medical Sciences, Wardha, IND

**Keywords:** chronic alcoholism, physiotherapy intervention, rehabilitation, physiotherapy, loculated pleural effusion

## Abstract

Pleural effusion is the accumulation of extra fluid between the layers of the pleura outside the lungs, also known as water on the lungs. Pleura are thin membranes that lubricate and aid breathing by lining the lungs and the inside of the chest cavity. The pleural space typically contains only a few teaspoons of watery fluid, which enables the lungs to move easily inside the chest cavity when breathing. Several barriers limited the patient’s capacity to carry out daily activities successfully and efficiently. Loculated effusions are most frequently associated with diseases such as empyema, hemothorax, or tuberculosis that result in severe pleural inflammation. Hence, a physiotherapy program is started to help improve the patient’s symptoms. A 59-year-old male presented to the hospital with the chief complaint of left-sided chest pain, fever, and breathlessness. On the Modified Medical Research Council dyspnea scale, breathlessness was grade 3.

## Introduction

A thin layer of tissue that surrounds each lung and folds back to form the lining of the chest cavity is known as the pleura. An excessive buildup of fluid in the pleural space is known as pleural effusion. Because it may be connected to pleural or lung illnesses, as well as systemic disorders, it may present a diagnostic challenge to the treating doctor. Patients typically complain of dyspnea, initially brought on by exertion, a mostly dry cough, and pleuritic chest pain [1]. Identifying whether a pleural effusion is a transudate or an exudate is the first step in evaluating patients who have one. If the patient complies with Light’s requirements, an exudative effusion is identified. The infrequent transudate that is misclassified as an exudate using these parameters may be more accurately classified using the serum to pleural fluid protein or albumin gradients. Therapy for the patient’s underlying heart failure or cirrhosis should be focused on in the presence of a transudative effusion [2].

Loculated effusions are most frequently associated with diseases such as empyema, hemothorax, or tuberculosis that result in severe pleural inflammation [1]. There may occasionally be fluid loculation in the fissures or between the pleural layers in the context of pleuritis (visceral and parietal). Blood, pus, and exudative fluid are the most frequent culturing substances [3].

For patients with pleural effusion and chest drains, conventional chest physiotherapy and intermittent positive airway pressure breathing are frequently recommended. Optimized lung expansion made possible by the use of intermittent positive airway pressure hastens the removal of pleural effusion, shortens the time that patients experience chest drainage and respiratory system impairment, shortens their hospital stays, and lessens the likelihood of developing pulmonary complications [4].

After being diagnosed with loculated pleural effusion, the patient in this case was treated and rehabilitated with the hopes of seeing an improvement in his health. He was asked to go to the physical therapy department where a suitable, well-planned treatment regimen was developed for the patient.

## Case presentation

**Figure 1:**
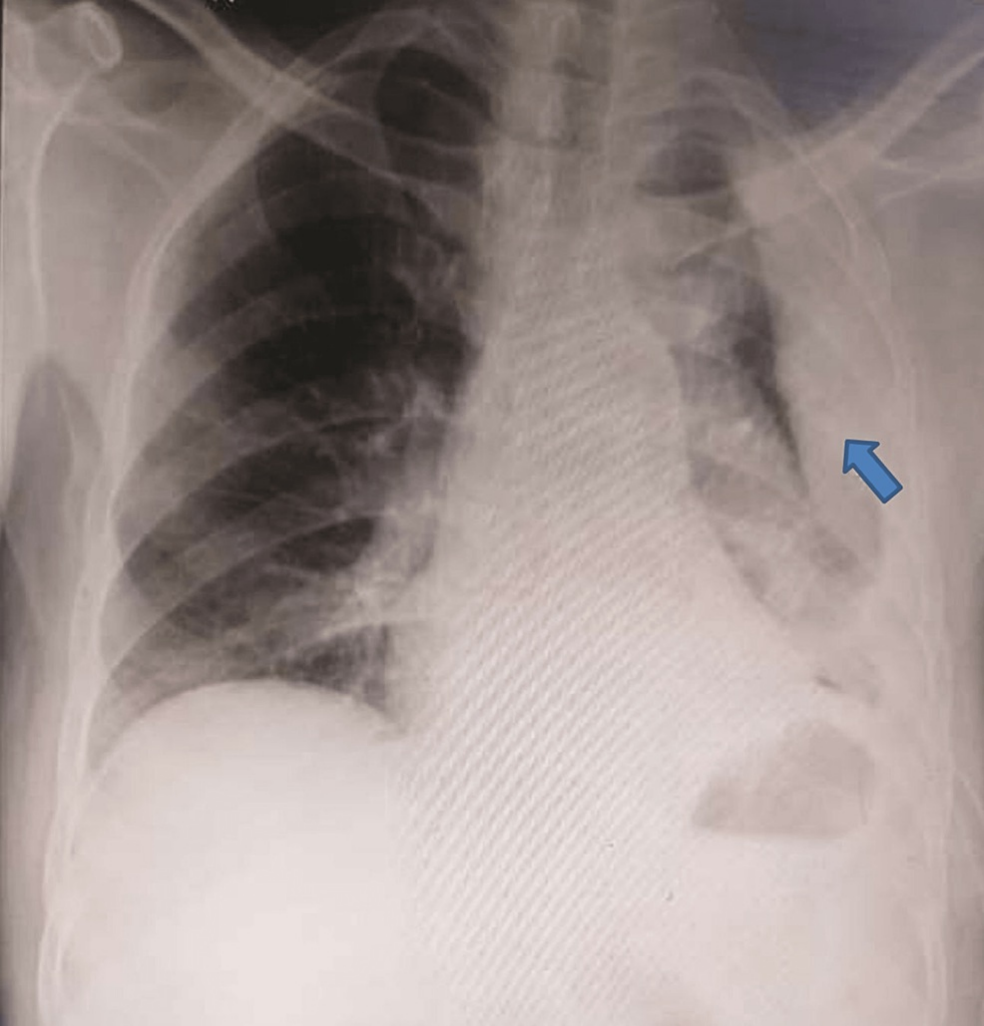
Chest X-ray of the patient. Obliteration of the left costophrenic angle with a large pleural-based dome-shaped opacity projecting into the lung was detected, with tracking along the costophrenic angle and lateral chest wall, indicating loculated pleural effusion.

A 59-year-old male patient who worked as a farmer was admitted to the hospital with complaints of left-sided chest pain and shortness of breath. According to the Modified Medical Research Council (MMRC) grading system, the patient had been in grade 3 for seven days. Regarding the chronic loculated pleural effusion, the patient was admitted for a month to a nearby hospital. An intercostal chest drain (ICD) had been in place for seven days when the patient was brought to the respiratory department. Computed tomography (CT) of the thorax CT was performed. He had a history of alcohol consumption for the past 10-15 years. The patient was active and was assessed by Berg’s balance scale for his balance, and no secondary complication was present due to alcohol consumption. The patient’s CT thorax revealed a significant left-sided pleural effusion with numerous loculated and encrusted collections, as well as underlying lung collapse, thickening of the pleura, and several enlarged lymph nodes. The patient’s X-ray is shown in Figure 1.

Therapeutic intervention

To stabilize and manage respiration, physiotherapy is crucial. Physiotherapists can efficiently increase secretion clearance and enhance ventilation by employing various techniques [5]. As the patient had an ICD, mild intervention was initiated, as shown in Table 1.

**Table 1:** Therapeutic intervention protocol.

Goals	Therapeutic intervention	Therapeutic protocol
Patient education	Educating the patient on the value of exercise. Obtaining the patient’s and his family’s cooperation and approval	Positioning, ambulation, and practical daily life activities were discussed with the patient and the caregiver
Bed mobility	Bedside sitting and transitions to beds are observed	The patient was shown how to roll over and sit by the bed. Positioning reduced the likelihood of developing bed sores, made drainage easier, and improved ventilation, which raised oxygen uptake
Retrain breathing pattern and improve dyspena	Exercises for controlled breathing were taught, such as diaphragmatic breathing and pursed lip breathing	The patient was instructed to carry out these breathing exercises 10 times, two to three times daily, to increase breathing efficiency
Improve lung volume	Exercises for thoracic expansion, shoulder flexion with a deep inhalation, and expiration with extension	It was advised to perform 10 repetitions in one set twice daily
Active range of motion for upper and lower limb	All range of motion activities taught to the patient	8–10 active repetitions each day for each joint. This preserved joint motion
Improve lung capacity	Shoulder flexion with deep inspiration and extension with expiration are thoracic expansion exercises. It uses incentive spirometers. Visual feedback is provided by three balls of varying colors, each indicating 600, 900, and 1,200 ccs	10 repetitions in one set twice a day at first; afterwards, twice a day, three to four times, for a total of 10 repetitions in two sets. The patient was initially instructed to perform spirometry two to three times daily; thereafter, it was advised that the patient perform spirometry every two hours
Mobilization	Halfway ambulation is initiated	Early mobilization helped the patient to improve functional residual capacity

Follow-up and outcome measures

The goal of pulmonary rehabilitation (PR), which is a comprehensive intervention based on a thorough patient assessment and patient-tailored therapies such as exercise training, education, and behavior change, is to improve the physical and mental health of patients with chronic respiratory diseases and to encourage long-term adherence to health-promoting behaviors [6]. Table 2 shows the outcome measures of pre and post-treatment.

**Table 2:** Outcome measures of the patient.

Outcome measure	Baseline	Discharge
Modified Medical Research Council dyspnea scale	3	2
Visual Analog Scale of dyspnea	5	3
Spirometer	Spirometer ball did not move	600 ccs

## Discussion

A loculated effusion, which might be lateral or lamellar, mediastinal, apical, subpulmonic, or fissural, causes atypical radiological results. Fissural loculations are biconvex opacities that resemble tumor tissue and dissolve after treatment. They are most commonly found in congestive heart failure [1].

Because of the difficulties surrounding treatment techniques and outcomes, treating loculated pleural effusion has always been difficult. The usefulness of urokinase as an intra-pleural fibrinolytic drug in the treatment of patients with loculated pleural effusion has been investigated. The presence of pleural fluid despite a radiologically verified properly positioned ICD can indicate numerous locules in the pleural cavity or tube obstruction due to increased fluid viscosity [7].

The study by da Conceição Dos Santos and Lunardi reported that patients with pleural effusion and chest drains should be frequently advised to use conventional chest physiotherapy and intermittent positive airway pressure breathing. The authors added that physiotherapy shortens hospital stays, lowers the frequency of pulmonary problems, and reduces the time spent with chest drainage and respiratory system impairment [4]. Another study by Valenza-Demet et al. also reported that patients with pleural effusion who also receive physiotherapy as part of their regular care have better spirometric and radiological results and shorter hospital stays [8].

## Conclusions

Our patient’s metrics improved with physical therapy rehabilitation. Patients gain from it by recovering more quickly. In this case, the patient was able to function following the rehabilitation with slight dyspnea. After rehabilitation, the patient’s breathlessness, spirometer, and drain were substantially better. Hence, the technique which was used helped the patient gain confidence.
